# Proteomic Identification of Pathways Responsible for the Estradiol Therapeutic Window in AD Animal Models

**DOI:** 10.3389/fncel.2019.00437

**Published:** 2019-10-15

**Authors:** Jie Cui, Jon Reed, Gogce Crynen, Ghania Ait-Ghezala, Fiona Crawford, Yong Shen, Rena Li

**Affiliations:** The Roskamp Institute, Sarasota, FL, United States

**Keywords:** Alzheimer’s disease, β-amyloid, cognition, hormone therapy, estrogen

## Abstract

Benefits and risks were reported for hormone therapy (HT) to prevent chronic disease, including Alzheimer’s disease (AD). While the Women’s Health Initiative (WHI) found no protective effect of HT on the cognitive function of women whose treatment was initiated far past the onset of menopause, other studies showed reduced risk of AD with midlife treatment, versus increased risk of AD with late treatment. These suggest a critical window during which estradiol must be administered to prevent cognitive decline and AD in women. Our published work supports this, by demonstrating that early and long-term estradiol treatment improves cognitive function and reduce Aβ accumulation in AD mouse models with estradiol deficiency, while there is no effect of late and short-term estradiol treatment on AD neuropathogenesis. However, little is known about the molecular mechanisms underlying the critical window and whether different protein networks are responsible for the brain estradiol deficiency-associated risk of AD in females. In this study, we used proteomics to identify target protein pathways that are activated during the estradiol therapeutic window in AD mouse model. Our results showed that different signaling pathways were involved in the regulatory effects of estradiol on MAP1A and hemoglobin α. Estradiol treatment increased the level of MAP1A through the phosphorylation of ERK1/2 and increased the level of hemoglobin α through the phosphorylation of AKT. This study has provided molecular insights into the “critical window” theory and identifies specific target proteins of therapeutic responsiveness that may lead to improved treatment strategies and optimal estradiol therapy.

## Introduction

Alzheimer’s disease (AD) is the most common form of dementia and most common neurodegenerative disease in the world. In the United States, around 5.4 million people are living with Alzheimer’s. The number is expected to almost triple to 16 million by 2050 (Hebert et al., 2003). In developed countries, AD is one of the most costly diseases to society, with an estimated cost of 20 trillion dollars by 2050. In light of the enormous costs associated with AD, a medication that delayed the onset of AD would significantly reduce this financial burden. Of the top 10 leading causes of death in the United States, AD is now the sixth (Murphy et al., 2015), and is the one which shows the greatest sex differences, the majority of AD patients being women (Kung et al., 2008). In the field of AD research, hormone therapy (HT) has received the greatest attention among all medications specifically prescribed for women. It has been shown that HT used at 65 years of age or older increases brain volume loss and the risk of dementia in women (Shumaker et al., 1998; Resnick et al., 2009), while HT used during the menopausal transition is thought to have protective effects on the brain (Sherwin, 2005). In addition, imaging studies showed that women receiving HT at early menopause for 4 years developed less AD plaques than women who were not treated (Kantarci et al., 2016). The critical window of HT (same as the timing hypothesis or the critical period hypothesis) states that the effects of HT depend on the timing of initiation of treatment with respect to age and/or the menopausal transition, and that optimal effects are evident with early initiation (Marder and Sano, 2000; Resnick and Henderson, 2002). There have been several observational studies or clinical trials providing support for the hypothesis, such as the Women’s Health Initiative Memory Study (WHIMS) (Shumaker et al., 2003; Shumaker et al., 2004), the Multi-Institutional Research in Alzheimer’s Genetic Epidemiology (MIRAGE) study (Henderson et al., 2005), the Cache County Study (Zandi et al., 2002), and the Kronos Early Estrogen Prevention Study (KEEPS) (Kantarci et al., 2018).

Our previous human and animal studies have also supported the “critical window” theory. First, we demonstrated that brain estradiol deficiency directly promotes early onset and increases severity of AD pathology in humans and animal models (Yue et al., 2005). Then, we showed that early estradiol treatment, rather than late treatment, can prevent amyloidgenesis in estradiol-deficient APP transgenic mice with ovariectomy (APP/OVX) (Li et al., 2013). Although there are extensive data supporting the critical window hypothesis, why the effects of HT on AD differ by age is unknown and the cellular mechanism(s) underlying the decreased response to estradiol with time are poorly understood.

In the present study, we tooka proteomics approach to identify the molecular landscape which defines the “critical window” in brain samples from APP and APP/OVXmice receiving early or late estradiol treatment. Proteins identified as being significantly modulated were then validated by western blot analysis. Our results showed that both hemoglobin α and MAP1A (Microtubule-associated protein 1A) were altered by estradiol treatments, but theMAP1A response in the mouse brain was specific to early, not late, treatment, suggesting that MAP1A expression is related to the “critical window” of HT on AD. We further studied the signaling pathways involved in the regulation of MAP1A and hemoglobin α, and found that different signaling pathways were involved in the regulatory effects of estradiol on MAP1A and hemoglobin α.

## Materials and Methods

### Animals

All mice were maintained in accordance with the NIH Guide for the Care and Use of Laboratory Animals. Wild type (WT) mice were from Jackson Laboratory (Bar Harbor, ME, United States) with C57BL/6 background. APP23 mice were from Novartis Pharma, Inc., Switzerland and were described in Sturchler-Pierrat et al. (1997). All mice were established on a C57BL/6J background and continuously back-crossed to C57BL/6J. Two estradiol deficiency mouse models (WT/OVX and APP/OVX) were generated by ovariectomizing WT and APP23 mice, respectively. At the age of 3 months, bilateral ovariectomies were performed on WT and APP23 mice, and sham surgery (the same surgical procedure except without removing the ovaries as a surgical control) was also performed on WT and APP23 mice. Therefore, there were four groups of mice: sham WT, sham APP23 (APP), ovariectomized WT (WT/OVX), ovariectomized APP23 (APP/OVX). All animal experiments were performed in compliance with a protocol approved by the Institutional Animal Care and Use Committee (IACUC) of Roskamp Institute.

### 17β-Estradiol Treatments

For early treatment, one week after the ovariectomy, the WT (*n* = 15) and APP (*n* = 15) mice were implanted subcutaneously with a sterilized 17β-estradiol (E2) pellet (1.7 mg or 18.9 μg/day). All pellets were made for 90-day release by the Innovative Research of America (Sarasota, FL, United States). Pellets were implanted every 3 months in order to maintain hormone levels until tissue harvest at the age of 12 months. A total of ten WT/OVX or 10 APP/OVX mice received the placebo pellet at the same age as control groups. For late treatment, at the age of 9 months, WT (*n* = 15) and APP mice (*n* = 15) wereimplanted with the same pellets at 9 months of age. A total of ten WT/OVX or APP/OVX mice received the placebo pellet at the same age as control groups. At the age of 12 months, all the mice were euthanatized and the brain tissues were harvested.

The total estradiol levels from brain or serum were as previously described (Yue et al., 2005).

### Cell Culture and Treatment

SH-SY5Y cells were cultured in 1:1 mixture of ATCC-formulated Eagle’s Minimum Essential Medium (ATCC, Catalog No. 30-2003), and F12 Medium, supplemented with 10% Fetal Bovine Serum (ATCC, Catalog No. 30-2020). For treatment studies, the SH-SY5Y cells were treated with 6 independent conditions, such as vehicle, 50 nM 17β-estradiol, 50 nM 17β-estradiol + 500 nM AKT inhibitor, 50 nM 17β-estradiol +1 μM MAPK inhibitor, AKT inhibitor alone or MAPK inhibitor alone. Each experiment was repeated at least three times. The inhibitors include AKT inhibitor (AKTi-1/2) (Abcam, ab142088) and ERK inhibitor (U0126) (Cell Signaling, #9903). The cells were treated with the inhibitors alone or after estradiol treatment. At end of the treatment, the cells were harvested in cell lysis buffer.

### Proteomic Analysis

A three-part fractionation was used to decrease sample complexity prior to tryptic digestion and LC-MS analysis. As shown in Supplementary Figure S1, frozen brain hemisphere was homogenized in modified phosphate buffered saline (NaCl concentration = 1M) with Halt Protease and Phosphatase Inhibitor Cocktail (PPIC) on ice in 10 s bursts. Following centrifugation at 20,000 × *g* at 4°C for 10 min the supernatant was mixed with chilled methanol (MeOH) for protein precipitation, while the pelleted material was saved and stored at −80°C as the “membrane” fraction for future use. The supernatants were incubated with chilled MeOH on ice for 30 min followed by centrifugation. All materials were then re-suspended in 20 mM triethylammonium bicarbonate (TEAB), pH 8.0 (Sigma Aldrich), 0.5% w/v sodium deoxycholate (SDC) via bath sonication. A process aliquot was taken to determine protein concentration using the bicinchoninic acid (BCA) assay (Pierce) and verified by SDS-PAGE and staining with Sypro Ruby. Samples were then prepared for tryptic digestion and labeling with Tandem Mass Tag (TMT) reagents (Thermo Fisher Scientific), 50 μg of each sample was then precipitated in four volumes of chilled acetone to concentrate and de-salt the material.

#### Tandem Mass Tag Labeling

As shown in Supplementary Figure S1, briefly, we employed a multiplexed isobaric labeling strategy to allow for simultaneous identification and quantification of proteins from multiple biological samples. Ten-plex TMT labeling kits (Thermo Fisher Scientific, Branchburg, NJ, United States) was used for analyses of protein samples from WT and APP23 mouse models, respectively. This format allowed for genotypes, time post-treatment and treatment groups to be analyzed within the same batch [early estrogen replacement treatment (ERT) included APP23/OVX + ERT, APP23/OVX + Placebo, WT/OVX + ERT, WT/OVX + placebo; lateERT had similar treatment group as for the early ERT except starting ERT at later age]. In addition, we had two additional group APP23 and WT that did not receive ovariectomy for a total of 10 group of mice. All samples and isobaric label tags were handled blind to the experimenter. Each TMT label was dissolved in 20 μl of acetonitrile solution. Ten microliters of digested protein from each sample were taken to dryness in a vacuum centrifuge. Twenty microliters aliquots of each label were also dried down in the speed vacuum and subsequently re-suspended in 25 mM TEAB in acetonitrile solution. Re-suspended labels were added to dried protein samples, and allowed to incubate for 1 h at room temperature, and the reactions quenched with addition of formic acid to a final concentration of 1% v/v. Labeled samples were pooled and subsequently taken to dryness in a vacuum centrifuge to remove acetonitrile prior to sample cleanup.

#### Sample Clean Up

Residual SDC and TEAB were removed from the samples as follows. The pooled, dried TMT-labeled protein samples were re-suspended in 100 μl of 1% formic acid in water, and centrifuged at 20,000 × *g* RCF for 1 min to remove the precipitated SDC. The supernatants were collected in new tubes, into which 400 μl of ethyl acetate was added, followed by vortexing to partition the residual SDC into the organic (upper) layer. Samples were then centrifuged at 20,000 × *g* RCF for 30 s, and upper organic layers were discarded. This was repeated three times, and the final lower phase was taken to dryness in the speed vacuum. Dried samples were re-suspended in 100 μl of 0.1% formic acid. Pooled TMT-labeled samples were concentrated and de-salted using C18 reversed phase ZipTips according to manufacturer’s protocol. ZipTip eluates were re-suspended in 20 μl of 0.1% formic acid, transferred into an auto-sampler vial, and analyzed by nano-UPLC MS on a Q-Exactive Orbitrap instrument (Thermo Fisher Scientific).

#### Chromatography and Mass Spectrometry Methods (LC-MS/MS)

Samples were analyzed by LC-MS/MS (Q-Exactive) as previously described (Abdullah et al., 2011; Zakirova et al., 2017). Data dependent acquisition (DDA) settings for the experiments were as follows: full-scan MS resolution = 140,000 full width at half maximum at 200 m/z, full-scan range = 380–1250 m/z, isolation width = 1.2 m/z, higher energy C-trap dissociation relative collision energy = 29, a minimum m/z setting of 100 m/z was used for all MS^2^ spectra, MS^2^ resolution = 17,500, dynamic exclusion = 180 s, and a Top 15 high/low duty cycle was used for precursor ion selection. These settings, particularly the narrow isolation window and the ultra-long gradient, were used to minimize the deleterious effects on quantitative accuracy that result from co-isolation of isobaric precursors without resorting to MS^3^-based methods.

### Data Processing and Statistical Analysis of Proteomics Data

Samples were analyzed by a Q Exactive benchtop LC-MS/MS (Thermo Fisher Scientific). PMI Preview software (PMI Software, Dublin, Ireland) was used to survey the data files and, if necessary, to add other modifications to the search criteria. Also, Preview results were used to choose the precursor and fragment ion mass tolerances (4 ppm, 0.02 Da, respectively) as well as dynamic modifications. The following settings were used to search the data using SEQUEST and BYONIC as the search algorithms, and Uniprot mouse database (02/2016). Dynamic modifications – Oxidation/ + 15.995 Da (M), Methyl/ + 14.016 Da (E), Deamidated/ + 0.984 Da (N, Q), static modifications of TMT6plex/ + 229.163 Da (N-Terminus, K), and carbamidomethyl + 57.021 (C). Only unique peptides were considered for quantification purposes. For SEQUEST, the Percolator feature of Proteome Discoverer, and for Byonic, the target-decoy feature, were used to set a false discovery rate (FDR) of 0.01 and peptides passing this cutoff value were exported to JMP 8.0.2 software (SAS Institute, Cary, NC, United States) for data cleaning and statistical analysis. Only unique peptides were considered for quantification purposes. Proteins identified with two or more peptides were used for the quantitative analysis. The ratios APP23/WT and of early treatment/late treatment were calculated for each condition after ln transformation of the raw ion counts. The ratios were normalized by central tendency normalization where medians were used. After the median of a peptide from multiple fractions was calculated, one sample *t*-test was used to test if the sample mean was equal to zero. The multiple-testing correction was applied to identify a “top tier” of significant proteins and prevent identification of false positives at a FDR of 5%. After the median per peptide sequence from multiple fractions were calculated, mixed model ANOVA was used to test for significant proteins changing overtime within each group.

### IPA Analysis

Datasets of significantly modulated proteins were uploaded to the Ingenuity^®^ Pathway Analysis software (IPA, Ingenuity Systems) in order to map the proteins onto known networks of protein interactions to ascertain the functional significance of APP and/or WT dependent changes in protein expression in each experimental paradigm (Kramer et al., 2014). In IPA the uploaded protein lists are assigned to established molecular pathways (“Canonical pathways”) and biological functions in the knowledgebase. The Core analysis settings were; Ingenuity Knowledge base as reference set, maximum number of molecules per network was 35, maximum number of networks for analysis was 25. Only experimentally observed knowledge was used. All species, data sources, tissues/cell lines included at the time of the analysis in IPA was considered. Core analysis identified the canonical pathways that were shown to be significantly modulated in response to treatment (early, late) or genotype (APP23, WT) as a result of significant modulation of proteins represented in those pathways. The significance of the association between the data set and the canonical pathway was measured in two ways. (1) For each canonical pathway a ratio of the number of molecules from the data set that map to that pathway divided by the total number of molecules in that canonical pathway is displayed. (2) Fisher’s exact test was used to calculate a *p*-value determining the probability that the association between the proteins in the dataset and the canonical pathway is explained by chance alone. *P*-values lower than 0.05 were considered significant.

### Western Blot

The mouse hemisphere was homogenized in mammalian protein extraction reagent (M-PER, Thermo Fisher Scientific, #78503) supplemented with Halt protease and phosphatase inhibitor single-use cocktail (Thermo Fisher Scientific, #78442). After centrifuging at 17,000 × *g* for 20 min, the supernatants were denatured and proteins were separated on 8% or 10% SDS-PAGE following by transfer to Nitrocellulose Membrane (Bio-Rad Laboratories, # 162-0112) at 90 mA, overnight at 4°C. Then the membranes were blocked with 5% dry milk dissolved in tris buffer saline (TBS). The membranes were incubated overnight with primary antibodies followed by the corresponding HRP-conjugated secondary antibodies (Santa-Cruz Biotechnology, #SC-2004 and #SC-2055). The primary antibodies included Goat anti-NBEA antibody (NOVUS BIOLOGICALS, #NB100-55385), Goat anti-Hemoglobin α Antibody (Santa Cruz, #sc-31333), Goat anti-Hemoglobin β Antibody (Santa Cruz, #sc-31116), Rabbit anti-neuroglobin antibody (Santa Cruz, #sc-30144), Mouse anti-MAP1 Antibody (EMD Millipore, #MAB362), mouse anti-β-actin antibody (Sigma Aldrich, #A1978), Rabbit anti-STIP1 antibody (Abcam, #ab126724), Rabbit Anti-BASP1 antibody (Abcam, #ab103315), Rabbit Anti-Fatty Acid Synthase antibody (Abcam, #ab22759), Rabbit Anti-Plectin antibody (Abcam, #ab83497), Rabbit Anti-NF-L antibody (Novus Biologicals, #NB300-131) and Rabbit Anti-MAP6 Antibody (LifeSpan BioSciences, #LS-C135766). Pierce ECL Western Blotting Substrate (Thermo, #32106) was used to develop the chemiluminescent image which was detected and captured by ChemiDoc XRS (Bio-Rad Laboratories) and the densitometry of the protein signals was measured using Quantity One software (Version 4.6.0, Bio-Rad Laboratories). The ratio of protein signals versus corresponding β-actin signal was calculated and expressed as density folds of the experimental group ratio to that of vehicle group, accordingly. All the western blots were repeated at least three times and the figures represent one of the typical results.

## Results

### Identification of Early or Late ERT Responding Proteins by Proteomics

**FIGURE 1:**
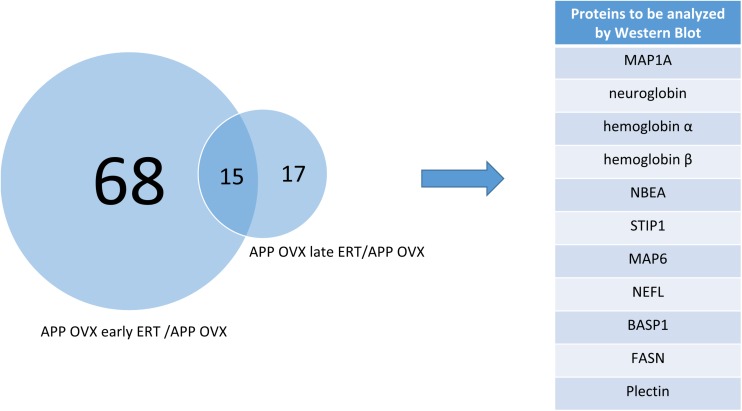
Venn diagram showing the overlap of significantly regulated proteins across two different comparisons, APP OVX early ERT/APP OVX and APP OVX late ERT/APP OVX.

**FIGURE 2:**
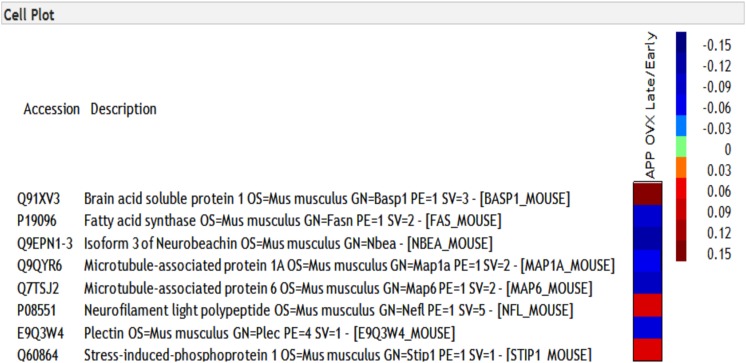
Heat map of eight proteins that were differentially regulated across early and late treatment groups in APP transgenic animals. The numbers presented in heatmap are the log of early/late ERT ratios.

To determine the molecular landscape which defines the “critical window” of estradiol therapy, we used proteomics approaches to identify the proteins that are differently modulated in response to early and late estradiol treatment in the brains of 12 months old APP/OVX mice. A total of 23724 PSMs (peptide spectrum matches), and 2233 non-redundant protein groups were identified. Only 1061 proteins that passed the criteria of being quantified in all MS experiments were included in the analysis. One sample *t*-test was used to identify proteins that were differentially expressed across different comparison groups, namely late ERT vs. early ERT; early ERT vs. no ERT; late ERT vs. no ERT, by testing if the ratio of interest was different from zero. As shown in Figure 1, 83 proteins were significantly modulated when comparing APP OVX Early treatment with APP OVX no treatment; and 32 were significantly modulated when comparing APP OVX Late treatment with APP OVX no treatment. There is an overlap of 15 proteins between these two groups. Among the 15 proteins, 8 proteins were significantly differently modulated between early and late treatment (Figure 1). The eight proteins that were differentially expressed across early and late ERT are shown in Figure 2.

### MAP1A (Microtubule-Associated Protein 1A) Is a Candidate Responsive Protein for the “Critical Window” of Estradiol Treatments

**FIGURE 3:**
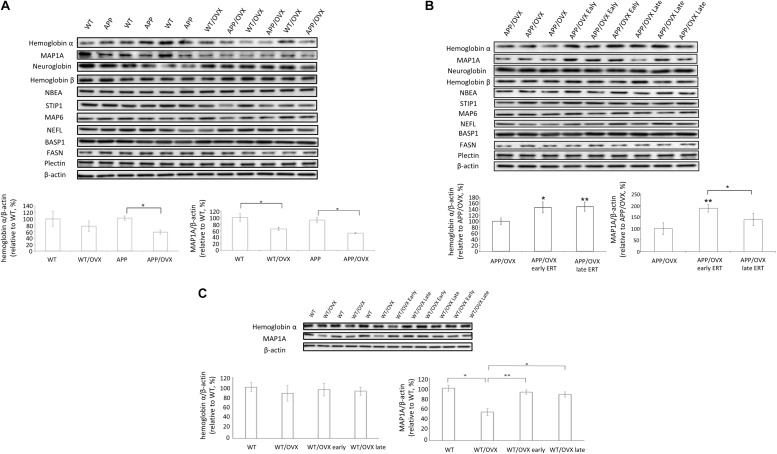
Western blot analysis of proteins identified in proteomic analysis. **(A)** The levels of hemoglobin α, MAP1A, neuroglobin, hemoglobin β, NBEA, STIP1, MAP6, NEFL, BASP1, FASN, and Plectin were detected in the brain lysates from WT, WT/OVX, APP, and APP/OVX mice, with β-actin as a loading control. **(B)** The levels of hemoglobin α, MAP1A, neuroglobin, hemoglobin β, NBEA, STIP1, MAP6, NEFL, BASP1, FASN, and Plectin were detected in the brain lysates from APP/OVX, APP/OVX early treatment group and APP/OVX late treatment group, with β-actin as a loading control. **(C)** The levels of hemoglobin α and MAP1A were detected in the brain lysates from WT, WT/OVX, WT/OVX early treatment group and WT/OVX late treatment group, with β-actin as a loading control. ^∗^Indicates P < 0.05 compared to APP/OVX or between the designated two groups. ^∗∗^Means P < 0.01 compared to APP/OVX or designated group as indicated.

Based on the proteomic findings, we further performed western blots to study these proteins related to ERT, including MAP1A, neuroglobin, hemoglobin α, hemoglobin β, NBEA, STIP1, MAP6, NEFL, BASP1, FASN, and Plectin, as shown in Figure 1. Our data showed that, in APP mice, OVX significantly downregulated the level of both hemoglobin α and MAP1A, suggesting the potential regulatory effects of 17β-estradiol on recovering levels of hemoglobin α and MAP1A. While in WT mice, OVX only downregulated the level of MAP1A, not hemoglobin α (Figure 3A). However, OVX did not change the level of other proteins (neuroglobin, hemoglobin β, NBEA, STIP1, MAP6, NEFL, BASP1, FASN, and Plectin) in either WT or APP mice (Figure 3A and Supplementary Figure S2). To further investigate whether early and lateE2 treatment could rescue OVX-induced downregulation of target proteins, western blots were performed on brain homogenates from APP/OVX, APP/OVX early treatment and APP/OVX late treatment groups. As shown in Figure 3B and Supplementary Figure S3, in APP mice, early E2 treatment rescued OVX-induced downregulation of hemoglobin α and MAP1A, while late E2 treatment only rescued OVX-induced downregulation of hemoglobin α, suggesting MAP1A is a candidate responsive protein for the “critical window” of estradiol treatment. Meanwhile western blots were also carried out on the genotype control groups including WT, WT/OVX, WT/OVX early treatment and WT/OVX late treatment. Interestingly, in contrast to the APP mice, in the WT mice both early and late treatment rescued the OVX-induced downregulation of MAP1A (Figure 3C). Furthermore, OVX did not induce any change of hemoglobin α level in WT mice (Figure 3C).

### Signaling Pathway Responsible for ERT-Related Proteins

Several signaling pathways have been reported to regulate the levels of hemoglobin α or MAP1A, such as PI3K/AKT, CaMK, CDK2, PKA, and MAPK. In this study, we used different inhibitors of signaling pathways to identify the connection between E2 and the target proteins.

**FIGURE 4:**
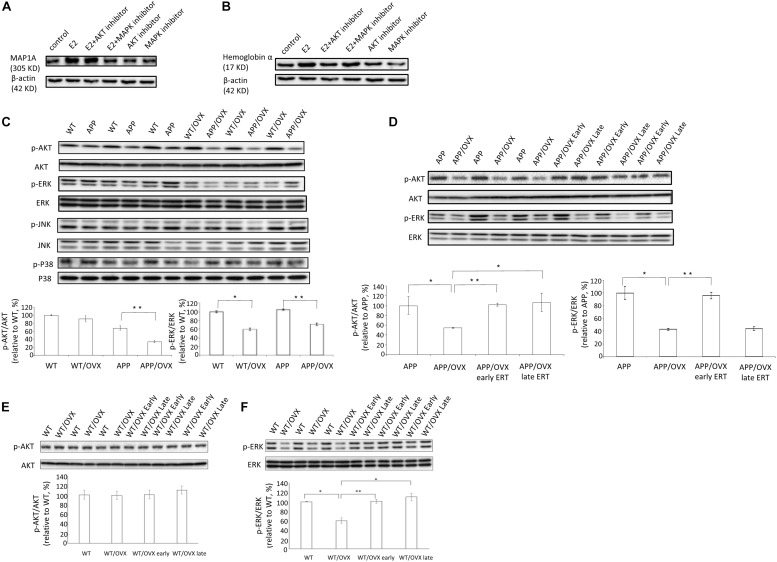
MAPK pathway and AKT pathway were involved in the regulatory effects of estrogen on MAP1A and hemoglobin α, respectively. **(A,B)** The levels of MAP1A and hemoglobin α were detected in SY5Y cell lysates, treated with AKT inhibitor or MAPK inhibitor, with β-actin as a loading control. **(C)** The phosphorylation status of AKT, ERK, JNK, and P38 were detected by western blot in the brain lysates from WT, WT/OVX, APP, and APP/OVX mice. **(D)** The phosphorylation status of AKT and ERK were detected by western blot in the brain lysates from APP, APP/OVX, APP/OVX early treatment group and APP/OVX late treatment group. **(E,F)** The phosphorylation status of AKT and ERK were detected by western blot in the brain lysates from WT, WT/OVX, WT/OVX early treatment group and WT/OVX late treatment group. ^∗^Indicates P < 0.05 compared to APP/OVX or between the designated two groups. ^∗∗^Means P < 0.01 compared to designated group as indicated.

Results from cell investigations showed that, among all the inhibitors tested, ERK inhibitor blocked E2-induced upregulation of MAP1A, suggesting the MAPK pathway might be responsible for the E2 effects on MAP1A (Figure 4A). For hemoglobin a, we found that AKT inhibitors can block E2-induced upregulation of hemoglobin α, indicating the AKT pathway might be responsible for the hemoglobin α response (Figure 4B). Based on this, to investigate the effects of OVX on the activation of different signaling pathways, the phosphorylation status of AKT, ERK, JNK and P38 were examined in APP and APP/OVX mice, with WT and WT/OVX mice as genotype controls. Our data showed that, in APP mice, OVX significantly downregulated both the level of AKT phosphorylation and that of ERK phosphorylation. In WT mice, OVX only downregulated the level of ERK phosphorylation (Figures 4E,F). However, the phosphorylation status of JNK and P38 were not affected by OVX in either WT or APP mice (Figure 4C and Supplementary Figure S4). We further examined the effects of early treatment as well as late treatment on OVX-induced downregulation of p-AKT and p-ERK in APP/OVX mice. As shown in Figure 4D, early treatment rescued OVX-induced downregulation of p-AKT and p-ERK. However, late treatment only rescued OVX-induced downregulation of p-AKT. The effects of OVX, early treatment as well as late treatment on ERK and AKT phosphorylation have good correlation with their effects on MAP1A and hemoglobin α, respectively, which strongly supporting that the MAPK pathway is responsible for E2 effects on MAP1A while the AKT pathway is responsible for the hemoglobin α effects.

## Discussion

The critical window hypothesis of HT and cognitive function states that the effects of HT depend on the timing of initiation with respect to age and/or the menopausal transition and that optimal effects are evident with early initiation.

Using proteomic approaches, we searched for the molecular landscape which defines the “critical window” for HT. We found that 83 proteins were significantly regulated in APP/OVX early ERT vs. APP/OVX and 32 proteins were significantly regulated in APP/OVX late ERT vs. APP/OVX. Among these proteins, there were 15 proteins that were overlapped between early and late ERT. Among these 15 proteins, only 8 of them were significantly differently regulated between early and late ERT.

Western blot analysis of brain homogenates showed that OVX appeared to induce the downregulation of hemoglobin α in only APP mice, not WT mice, whereas OVX induced the downregulation of MAP1A in both WT mice and APP mice. Meanwhile, both early and late ERT rescued OVX-induced downregulation of hemoglobin α in APP mice. However, only early ERT, not late ERT, rescued OVX-induced downregulation of MAP1A in APP mice. These results indicate that MAP1A plays an important role in the critical window of HT. Furthermore, in WT mice, both early and late ERT rescued OVX-induced downregulation of MAP1A, indicating the influence of genotype (APP or WT) on whether there is a “critical window” for the regulatory effects of 17β-estradiol treatment on MAP1A levels in the mouse brain.

MAP1A is a microtubule stabilizing protein that belongs to the microtubule-associated protein family. The proteins of this family are thought to be involved in microtubule assembly and responsible for the polymerization, stabilization and dynamics of the microtubule network, which is essential in neurogenesis. MAP1A is abundantly expressed in neurons, prominently in dendrites and to a lesser extent in cell bodies and axons (Riederer and Matus, 1985). It has been reported that MAP1A plays important roles in neurogenesis and is essential for the stabilization of microtubules during the late phase of postnatal development and involved in the differentiation of axons and dendrites as well as maintenance of the microtubule cytoskeleton in mature neurons (Matus, 1991). Recent evidences supports the notion that microtubule rearrangements may be proximate to neurite degeneration and deficits in episodic declarative memory, which is the earliest manifestation of AD (Faller et al., 2009). MAP1A potentially serves a key function in synaptic plasticity, as neuronal cultures transfected with MAP1A siRNA display inhibition of activity-induced dendritic branching and retraction of already existing branches (Szebenyi et al., 2005). Under physiological conditions, MAP1A is responsible for stabilization and dynamics of the microtubule networks. Under AD conditions, it has been reported that there is a time-dependent decrease of MAP1A levels, and loss of MAP1A function leads to synapse dysfunction and synapse loss in the hippocampus. Therefore, estradiol might prevent AD pathology through the improvement of synapse function mediated by MAP1A. Here, we showed that MAP1A is a candidate responsive protein which is associated with the critical window of AD treatment involving estradiol.

For hemoglobin α, it has been reported that lower levels of hemoglobin α is associated with cognition decline in older persons. Moreover, it has been reported that hemoglobin α has been identified as one of the biomarkers which is significantly decreased in AD (Chuang et al., 2012). Also, hemoglobin α has neuroprotective effects, probably through the elimination of Reactive Oxygen Species. Here, we are testing whether the OVX would result a regulation of hemoglobin α expression. If so, the whether the effect of OVX would be a direct effect or a compensatory effect. In the brain, there are three globin family members – hemoglobin α, hemoglobin β, and neuroglobin. Hemoglobin β is an isoform of hemoglobin α. Neuroglobin is a heme protein predominantly expressed in neurons and has neuroprotective effects. So we also included all the three globin family members in our WB experiments. Our results showed that, in estradiol-induced protective effects on AD, hemoglobin α might be a general regulator of AD pathology, not an age-sensitive one.

We also investigated the signaling pathways involved in the regulation of MAP1A and hemoglobin α, respectively, to reveal the molecular mechanisms behind the observation that only early estradiol treatment rescued MAP1A levels in APP mouse brain, while both early and late estradiol treatment rescued hemoglobin α levels in APP mouse brain as well as MAP1A levels in WT mouse brain. In detail, we found out that ERK inhibitor (U0126) blocked E2-induced upregulation of MAP1A, showing that the MAPK pathway was responsible for the E2 effects on MAP1A. And AKT inhibitor (AKTi-1/2) blocked E2-induced upregulation of hemoglobin α, showing that the AKT pathway was responsible for the hemoglobin α response.

**FIGURE 5:**
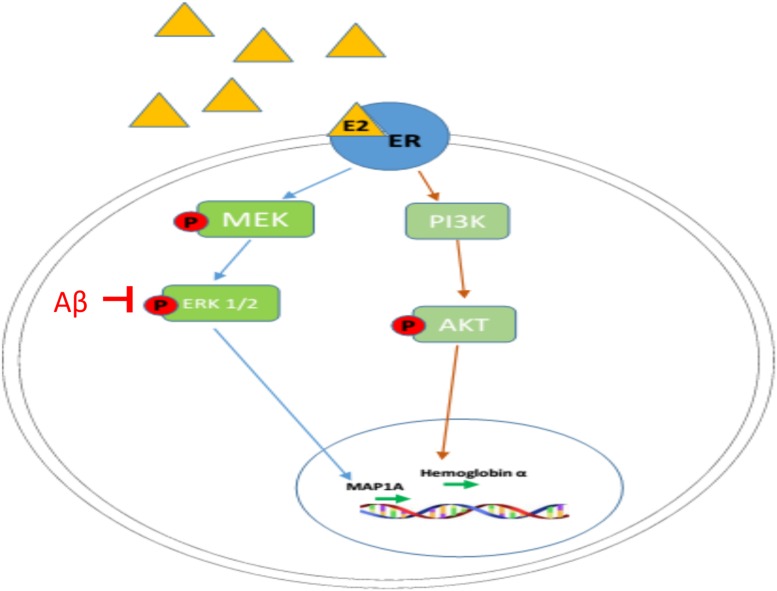
Mechanism of regulatory actions of E2 on MAP1A and hemoglobin α transcription. Estrogen (E2 shown as a yellow triangle) binds the estrogen receptor (ER shown as a blue oval) to form a complex. The E2-ER complex triggers the activation of MAPK pathway and PI3K/AKT pathway, leading to the upregulation of MAP1A and hemoglobin α, respectively.

It has been reported that the phosphorylation of ERK1/2 (activation of MAPK pathway) can be significantly inhibited by β-amyloid in organotypic hippocampal slice cultures and neuronal cell lines, as well as in AD animal models, as shown in Figure 5 (Daniels et al., 2001; Bell et al., 2004; Ma et al., 2007). Since E2-induced up-regulation of MAP1A is dependent on MAPK pathway activation (as shown in Figure 4A), the β-amyloid induced inhibition of MAPK pathway in the APP mice brain at older ages provide one possible explanation why only early, not late, estradiol treatment, can rescue estradiol deficiency-induced downregulation of p-ERK1/2 as well as MAP1A levels in the APP mice brain, while both early and late estradiol treatment can rescue the MAP1A levels in WT mice brain.

In addition to the “critical window” theory, there are also other hypothesized mechanisms which explain the contradiction of HT. The most famous one is the “healthy-cell bias” theory, which states that estradiol has protective effects in healthy neurons, even in aged cells undergoing some pathological changes, such as amyloid-related pathology, show acceleration in their demise when exposed to estradiol. This theory has been supported by cell culture experiments showing how healthy neurons benefit from an estradiol-enriched environment, although once neuronal cells start undergoing mitochondrial pathological change, estradiol can accelerate this pathogenic process, leading to cell death (Brinton, 2008). Several observational human studies confirmed the “healthy-cell bias” theory, showing that higher estradiol levels were associated with worse hippocampal atrophy and memory impairment (both early indicators of AD) in older women (den Heijer et al., 2003). While our results suggest that early and long-term usage of E2 and/or genistein may prevent AD pathologies in aged females via promoting synaptic plasticity, others reported that the mechanism for the closing of the therapeutic window may involve a shift in estradiol receptor (ER) expression (Foster, 2005, 2012; Bean et al., 2014).

In conclusion, while the critical window of HT for AD is very important and the underlying mechanisms for the critical window have been understudied. In our study, we used proteomics approach and identified specific target proteins of therapeutic responsiveness that may lead to improved treatment strategies and optimal estradiol therapy. This study has provided molecular insights into the “critical window” theory.

## Data Availability Statement

All datasets generated for this study are included in the manuscript/Supplementary Files.

## Ethics Statement

The animal study was reviewed and approved by the Roskamp Institute Institutional Animal Care and Use Committees.

## Author Contributions

JC and RL performed most of the animal experiments as well as the manuscript preparation. JR and GC performed the proteomics and data analysis. FC and GA-G provided help on methods and discussion for this project. YS and RL were responsible for the experimental design, results, and discussion as well as the manuscript preparation for publication.

## Conflict of Interest

The authors declare that the research was conducted in the absence of any commercial or financial relationships that could be construed as a potential conflict of interest.

## Supplementary Material

FIGURE S1 Proteomics workflow. **(A)** Depicting protein extraction, and schematic of proteomics experiments conducted using brain homogenates from WT, APP/OVX, WT OVX mice receiving estradiol treatment at early or late time point using TMT. **(B)** Methodologies.

FIGURE S2 The analysis for proteins in Figure 3A. **(A)** Neuroglobin. **(B)** Hemoglobin β. **(C)** NBEA. **(D)** STIP1. **(E)** MAP6. **(F)** NEFL. **(G)** BASP1. **(H)** FASN. **(I)** Plectin.

FIGURE S3 The analysis for proteins in Figure 3B. **(A)** Neuroglobin. **(B)** Hemoglobin β. **(C)** NBEA. **(D)** STIP1. **(E)** MAP6. **(F)** NEFL. **(G)** BASP1. **(H)** FASN. **(I)** Plectin.

FIGURE S4 The analysis for proteins in Figure 4. **(A)** MAP1A. **(B)** Hemoglobin α. **(C)** The ratio of p-JNK/JNK. **(D)** The ratio of p-p38/p38.
