# Supplementary figures and images for: Proteomic Identification of Pathways Responsible for the Estradiol Therapeutic Window in AD Animal Models
Source: Front Cell Neurosci. 2019 Oct 15;13:437. doi: 10.3389/fncel.2019.00437 (PMC6804529; doi:10.3389/fncel.2019.00437)

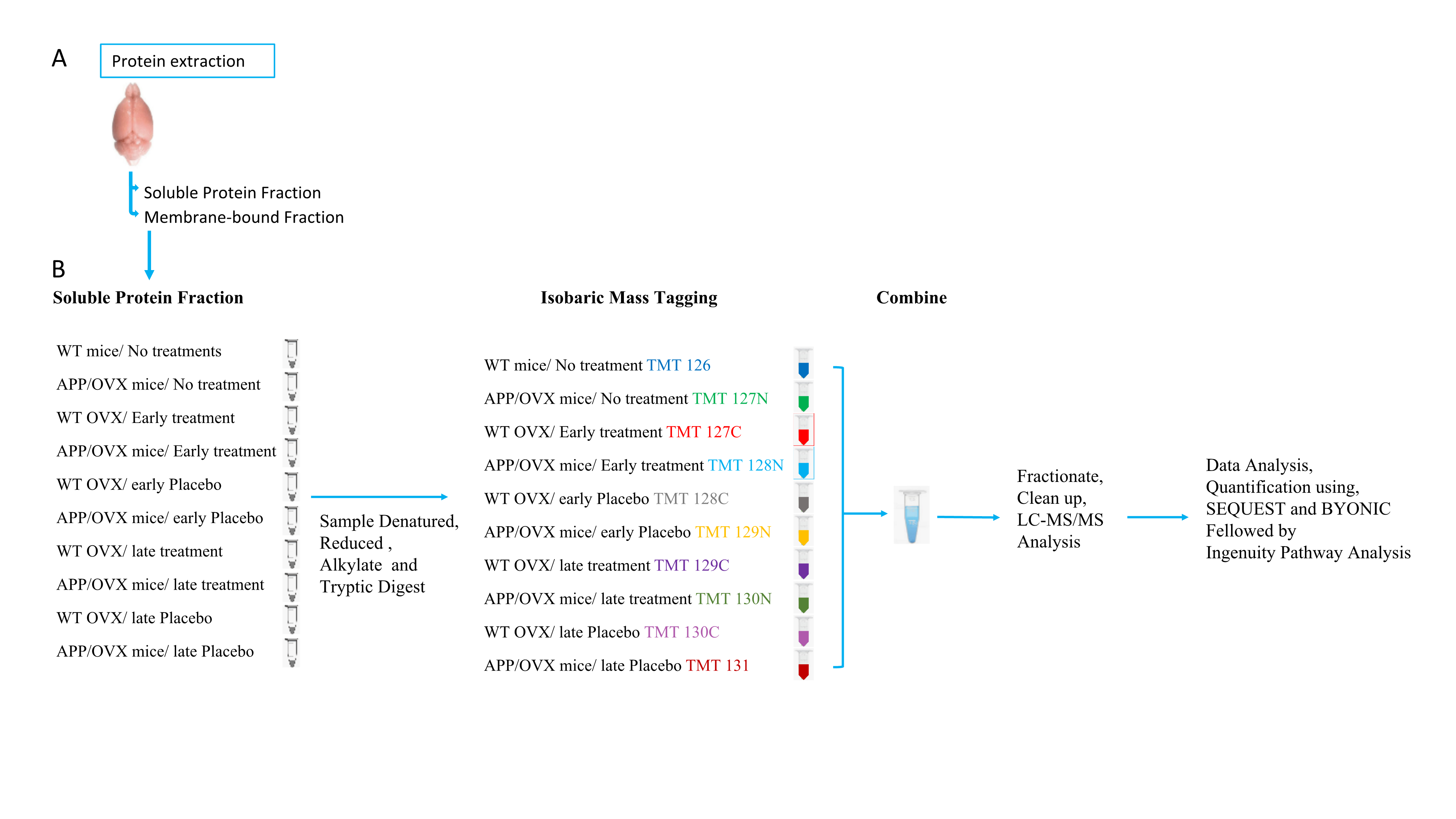

Supplement: FIGURE S1 — Proteomics workflow. (A) Depicting protein extraction, and schematic of proteomics experiments conducted using brain homogenates from WT, APP/OVX, WT OVX mice receiving estradiol treatment at early or late time point using TMT. (B) Methodologies. [file Image_1.TIFF]

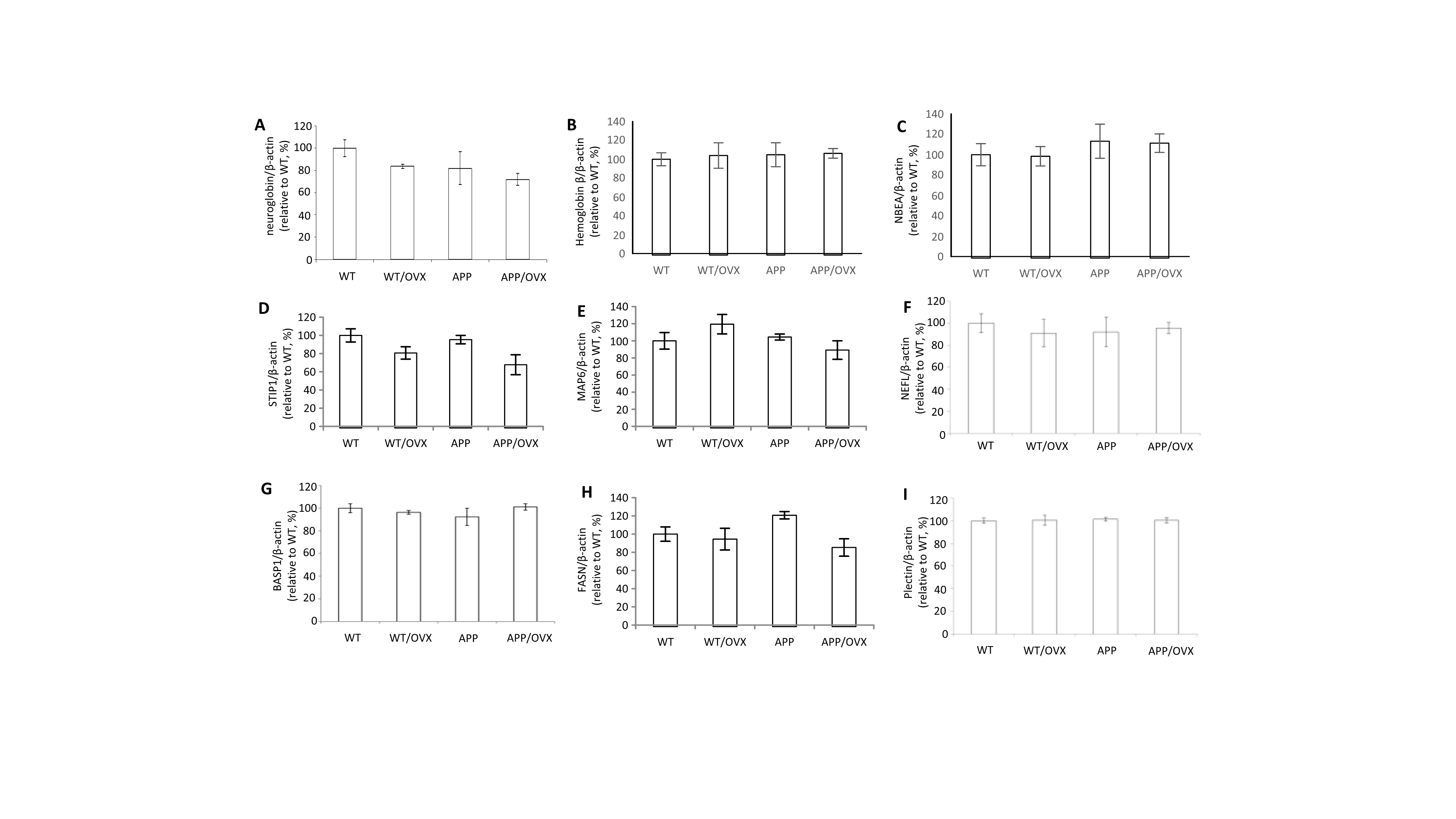

Supplement: FIGURE S2 — The analysis for proteins in Figure 3A. (A) Neuroglobin. (B) Hemoglobin β. (C) NBEA. (D) STIP1. (E) MAP6. (F) NEFL. (G) BASP1. (H) FASN. (I) Plectin. [file Image_2.TIFF]

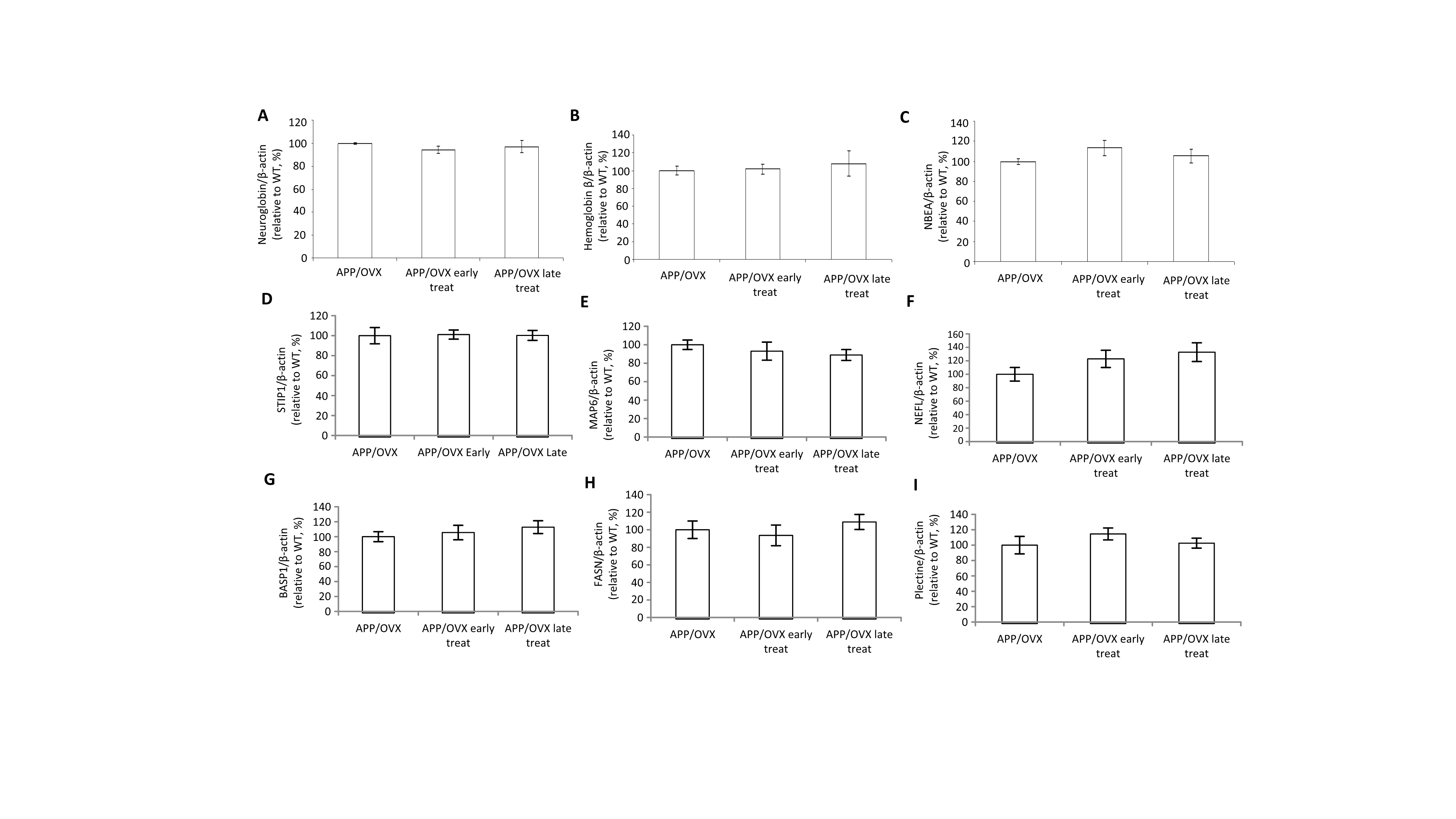

Supplement: FIGURE S3 — The analysis for proteins in Figure 3B. (A) Neuroglobin. (B) Hemoglobin β. (C) NBEA. (D) STIP1. (E) MAP6. (F) NEFL. (G) BASP1. (H) FASN. (I) Plectin. [file Image_3.TIFF]

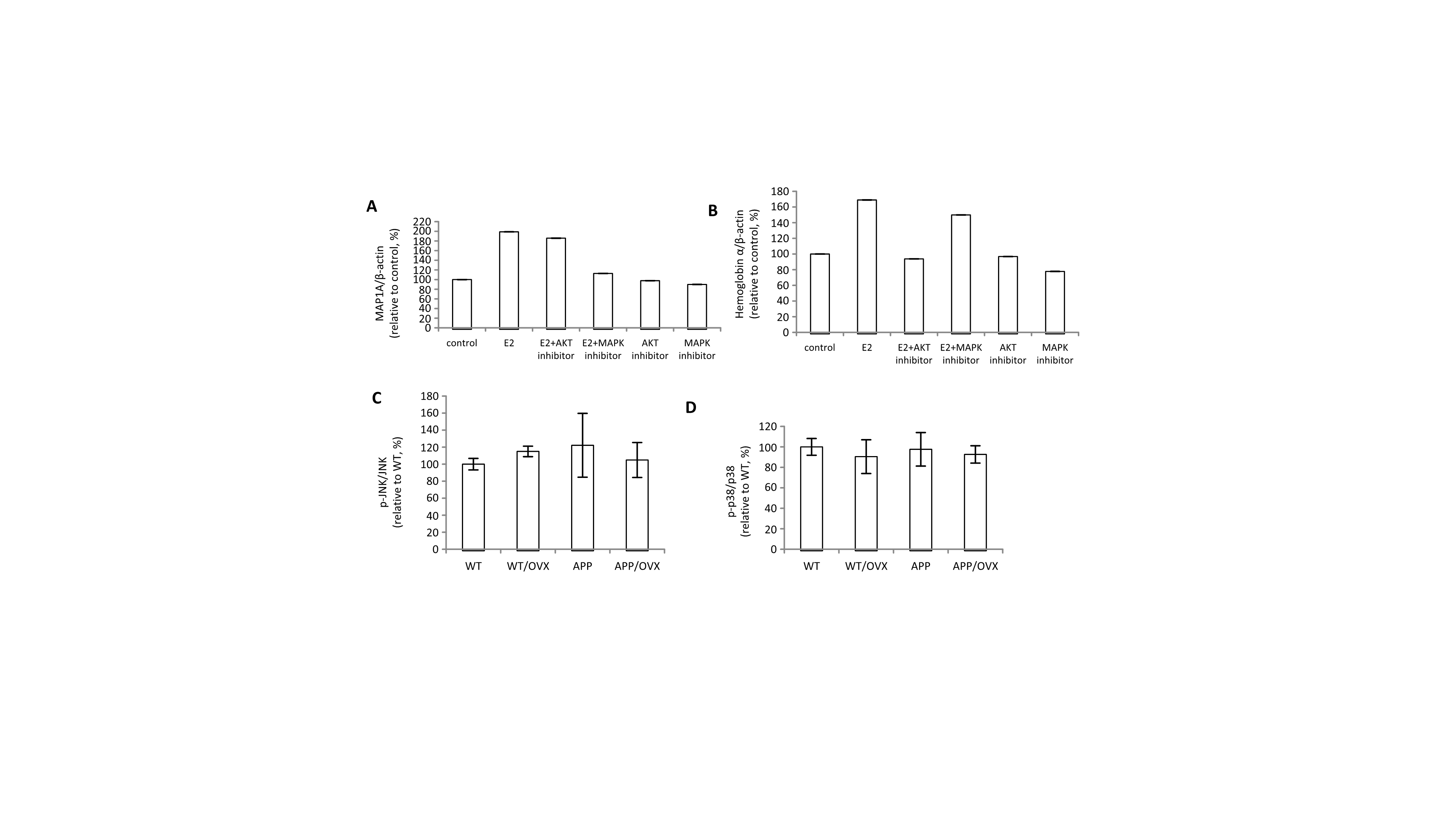

Supplement: FIGURE S4 — The analysis for proteins in Figure 4. (A) MAP1A. (B) Hemoglobin α. (C) The ratio of p-JNK/JNK. (D) The ratio of p-p38/p38. [file Image_4.TIFF]
